# Scratching the surface: the use of sheepskin parchment to deter textual erasure in early modern legal deeds

**DOI:** 10.1186/s40494-021-00503-6

**Published:** 2021-03-25

**Authors:** Sean Paul Doherty, Stuart Henderson, Sarah Fiddyment, Jonathan Finch, Matthew J. Collins

**Affiliations:** 1 Department of Archaeology, University of Exeter, Exeter, UK; 2 Department of Archaeology, University of York, York, UK; 3 McDonald Institute for Archaeological Research, University of Cambridge, Cambridge, UK; 4 Section for Evolutionary Genetics, GLOBE Institute, University of Copenhagen, Copenhagen, Denmark

**Keywords:** Parchment, Manuscripts, Proteomics, Biocodicology, Sheepskin, Legal deeds

## Abstract

Historic legal deeds are one of the most abundant resources in British archives, but also one of the most neglected. Despite the millions that survive, we know remarkably little about their manufacture, including the species of animal on which they were written. Here we present the species identification of 645 sixteenth–twentieth century skins via peptide mass fingerprinting (ZooMS), demonstrating the preferential use of sheepskin parchment. We argue that alongside their abundance and low cost, the use of sheepskins over those of other species was motivated by the increased visibility of fraudulent text erasure and modification afforded by the unique structure of their skin.

**Supplementary Information:**

The online version contains supplementary material available at 10.1186/s40494-021-00503-6.

## Introduction

By the late-sixteenth century, English common law was predominantly text-based, displacing oral tradition as the repository of legal precedent [1]. Deeds, wills and other legal instruments grew in significance amongst all social stations [2–4] as the burgeoning statute book^1^ increasingly necessitated formally executed documents through which an interest, right, property or obligation could be created, confirmed or transferred.

**Fig. 1:**
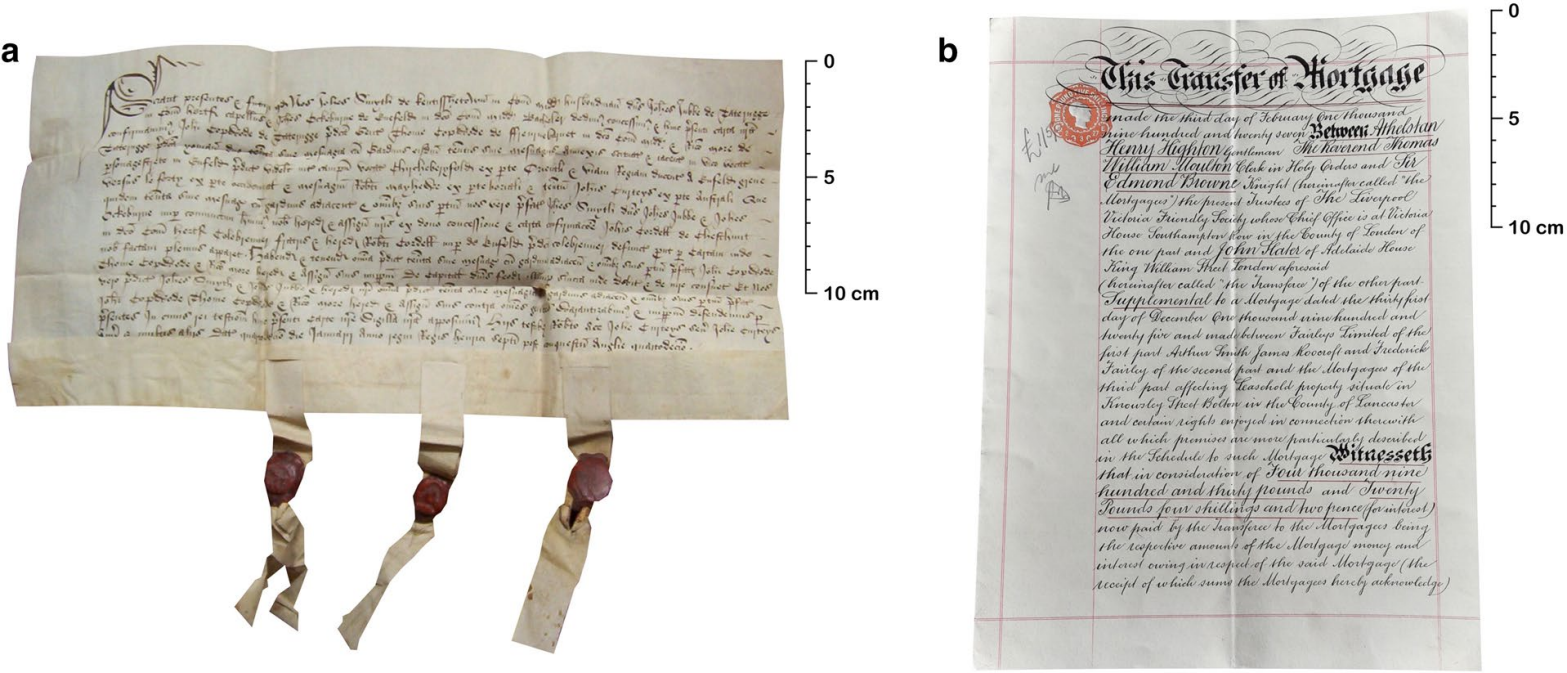
Deeds: **a** title deed concerning the ownership of land in Enfield, Middlesex, signed and sealed 15th January 1499 (sample DL035, photograph courtesy of Dave Lee); **b** mortgage between Athelstan H. Highton Esq., Rev. Thomas Moulton and Sir Edmund Browne on property in Bolton, Lancashire, signed and sealed 3rd February 1927 (sample EB04, photograph by Sean Paul Doherty)

Despite the permissibility^2^ and growing use of paper, deeds—legal documents concerning the ownership or tenure of tangible (land or buildings) and intangible (rights or privileges) property—remained principally handwritten on animal skin (Fig. 1) [5]. The continued use of skins, despite their significantly higher cost [6–8], is likely due to their greater durability than other writing media. The enhanced longevity afforded to text written on skin rather than paper was noted by contemporary theologians and jurists alike [9–12], echoing comments of the durability of skin over papyrus a millennia earlier [13].

Such is their durability that historic legal deeds are one of the most abundant resources in British archives; but they are also one of the most neglected [14, 15]. When viewed simply as a textual resource, they are often considered to be of limited historic or scholarly value due to the considerable proportion of text taken up by formulaic rubric. Many were discarded, burnt, or even repurposed into lamp shades during the twentieth century after the Land Registry Act of 1925 (15 & 16 Geo. 5. c.21) ceased the necessity of their retention [15–17]. We know remarkably little about the production of these commonplace legal documents. Uncertainty even remains over the animal species used, with deeds frequently catalogued as ‘vellum’ (etymologically meaning calfskin), ‘parchment’ (typically reserved for sheep or goatskin), or even more generally as ‘animal membrane’.

Species identification based upon observation of hair fibres and follicle patterns that survived the manufacturing process has suggested a potential preference of sheepskin for legal deeds across the thirteenth–nineteenth centuries [18, 19]. This method of identification relies on subjective identification by the user which can lead to misidentification [20], though Ryder’s conclusions have been supported more recently by genetic and proteomic analyses [20–22]. Yet, despite the quantities that survive, the corpus of identified material remains limited. Here we present the species identification of 645 legal deeds from the sixteenth to twentieth century using peptide mass fingerprinting (ZooMS) and explore the potential drivers behind the choice of animal.

## Materials and methods

Samples were obtained from 645 individual pages from a total of 477 deeds concerning property in England, Scotland and Wales (Table 1). Of the documents with multiple pages, each skin was of a size (> 70 × 50 cm) to indicate they came from a single animal. Each deed was engrossed with the day, month and year the agreement was signed. None had received any conservation treatment or presented any visual evidence for being a palimpsest (from the Ancient Greek ‘παλίμψηστος’ meaning ‘scraped again’), where the previous text has been erased and the parchment reused, as determined via gross examination. Physical samples (0.2 cm^2^) were removed from the edge of each leaf from areas devoid of any ink, pencil, stamp, glue or surface marking to avoid contamination.

**Table 1:** Collection information of deeds analysed

Collection	*n*	Date range (AD)	Collection information
Cheshire records office	15	1786–1813	Artificial collection of title deeds concerning property in Cheshire
Doherty collection	8	1913–1940	Artificial collection of title deeds concerning property across England and Wales
Hull history centre	38	1596–1969	Artificial collection of title deeds concerning property in the East Riding of Yorkshire
Lee collection	254	1499–1907	Artificial collection of title deeds concerning property across England, Wales and Scotland
Lincoln records office	9	1742–1907	Artificial collection of title deeds concerning property in Lincolnshire
Lord collection	50	1582–1893	Title deeds concerning Lower Winskill Farm, Settle, North Yorkshire
Tye collection	254	1650–1904	Artificial collection of title deeds concerning property in the City of London. Documents were discarded from the Sun Fire Office, London, company archives
Westminster city archives	1	1707	Title deed from the City of Westminster
Wills collection	16	1652–1790	Title deeds concerning property in Somerset

The artificial collections contain documents of different provenance, while the others have grown organically around a single property

Samples were placed in individual 1.5 ml microcentrifuge tubes, 75 μl of 0.05 M ammonium bicarbonate (NH_5_CO_3_) buffer added, along with 1 μl of porcine trypsin (0.47 μg/μl) (Promega, WI, USA) and incubated at 65 °C to gelatinise. After 4 h, 1 μl of trifluoroacetic acid (TFA) (5% vol/vol) was added to cease enzymatic digestion. The digest was desalted and purified using C_18_ solid-phase tips (Agilent ZipTip, CA, USA), and the peptides eluted in a final solution of 50 μl, 50% acetonitrile/0.1% TFA (vol/vol). 1 μl of eluted peptides was mixed on a ground steel plate with 1 μl of α-cyano-4-hydroxycinnamic acid matrix solution [1% in 50% ACN/0.1% TFA (vol/vol)] and allowed to co-crystallise. All samples were spotted in triplicate. Samples were analysed using a Bruker Ultraflex II (Bruker Daltonics, Bremen, Germany) MALDI-TOF instrument equipped with a Nd:YAG smart beam laser. Samples spectra were calibrated against an adjacent calibrant spot with six calibration peptides. The resulting mass spectra were analysed within mMass software (https://www.mmass.org) [23], and individual peptides manually identified according to published markers [24, 25].

## Results

**Fig. 2:**
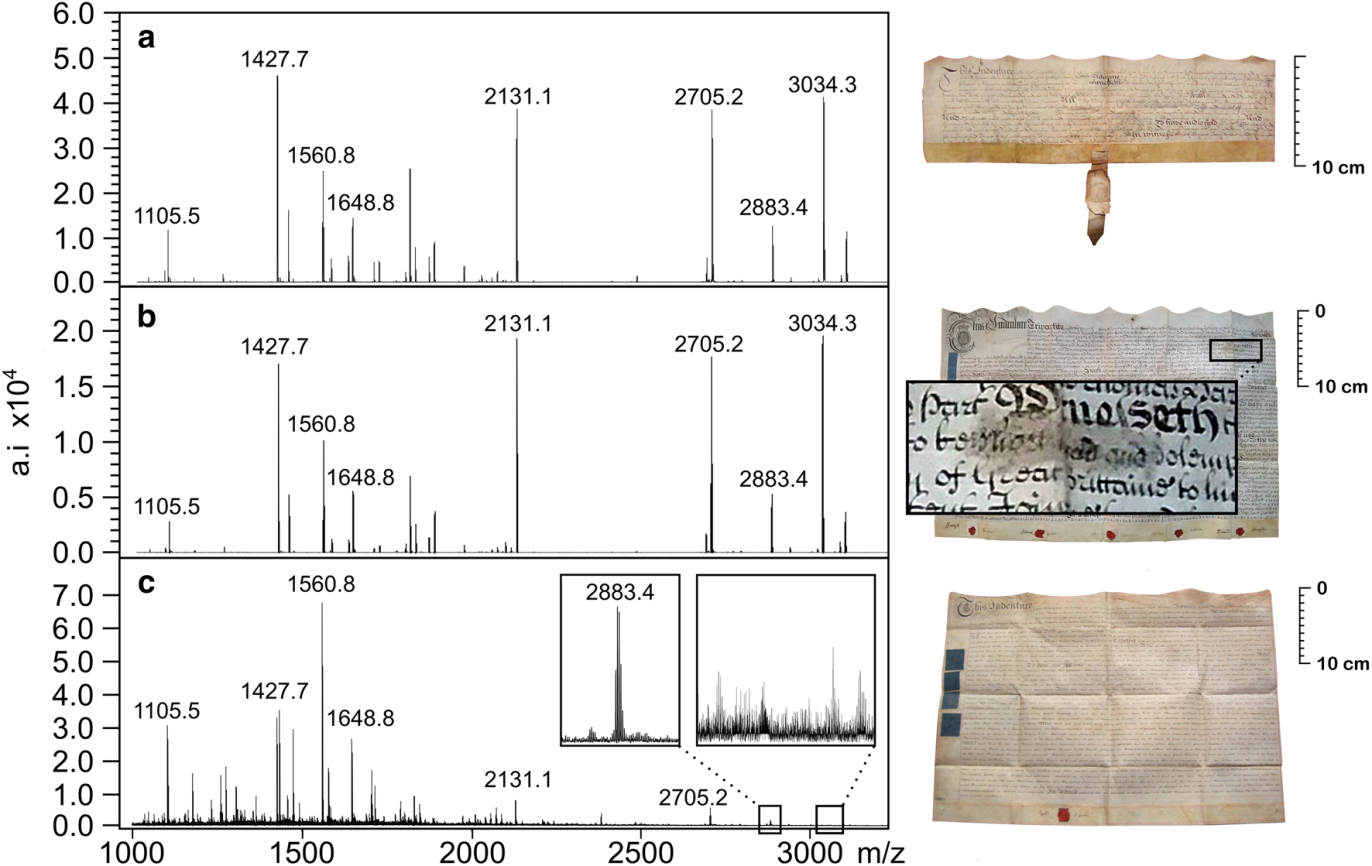
Peptide mass fingerprint from samples. **a** DL157, an indenture concerning property in Bennington, Hertfordshire, signed 20th May 1687. Identified as sheepskin based on diagnostic peptide markers, particularly at 3034.3 (m/z) [25]. **b** DL189, a tripartite marriage settlement signed 14th November 1737 in Atterton, Leicestershire. Identified as sheepskin based on diagnostic peptide markers*.* Note the visible erasure of text amending the intended date of the wedding. **c** DL149, an indenture concerning property in Knaresborough, North Yorkshire, signed 3rd June 1800. While the peptide marker at 2883.4 indicates it is either sheep or goatskin and not calfskin (*Bos taurus*) the absence of diagnostic peptide markers at 3034.3 (sheep) and 3094.3 (goat) [25] precludes separation of these species (photographs of deeds courtesy of Dave Lee)

All 645 samples were identified as animals of the* Bovidae* family, of which 622 (96.4%) were identified as sheep (*Ovis aries*). The remaining 23 (3.6%) could be classified as sheep or goat (*Capra aegagrus hircus*), but separation between the species was not possible due to the lack of diagnostic peptides (Fig. 2). Protein survival in parchment can be reduced via oxidation, hydrolysis and biological attack during storage [26, 27], and likely affected the presence of diagnostic peptides within these samples. These samples came from a range of collections and the absence of sufficient diagnostic peptides does not appear to be related to their age or storage location. This highlights the potential limitation of this biomolecular technique and the continued role that fibre and follicle analysis has in the identification of historic parchment. It is highly likely that most, if not all samples are sheepskin, but acknowledging the visual identification of goatskin by Ryder [18] in contemporary documents, the presence of this species can not be ruled out.

## Discussion

Although de Hamel [28] contends that neither the scribe or recipient knew nor cared what animal the parchment was made from, the evidence suggests otherwise; sheepskin parchment was preferentially selected over that of calf or goat for legal deeds, a selection which extends back to at least the thirteenth–fourteenth century in England, Wales and Ireland [18–20, 22, 29].

**Fig. 3:**
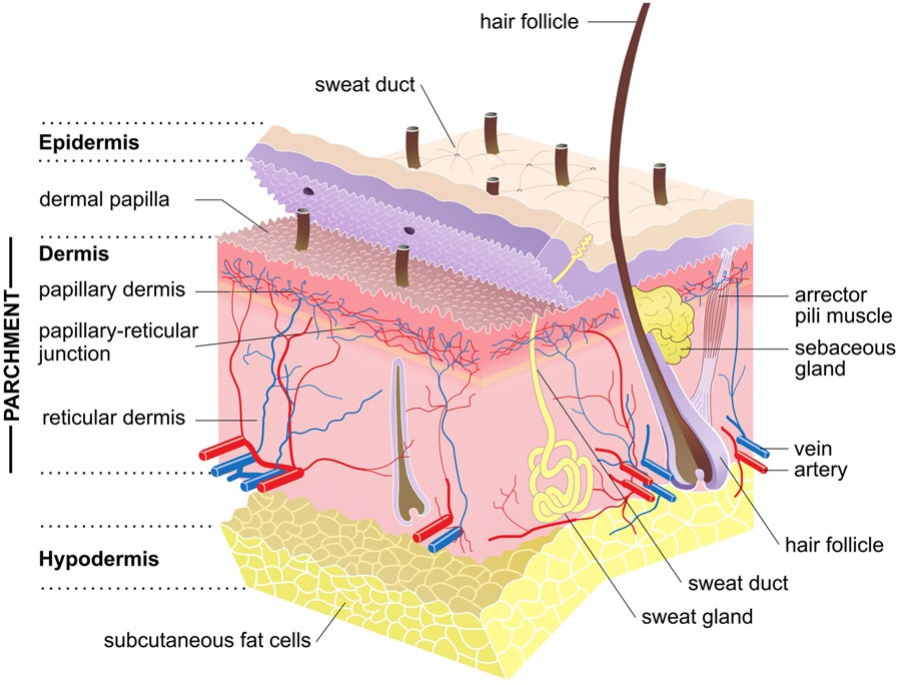
Structure of sheepskin and the layers typically present in parchment (Sean Paul Doherty, Wikimedia Commons)

The roots of this preference may lie in early efforts to impede the fraudulent modification of legal agreements after signing due to the increased visibility of erasure and text alterations afforded by sheepskin. Parchment is made from the dermis layer of skin, a layer divided into the fine dermal fibres of the upper *papillary dermis* and larger fibres of the lower *reticular dermis* (Fig. 3)*.* This intersection is characteristically weak in sheepskin due to the abrupt change in structure and the presence of cutaneous lipids which form within the *papillary-reticular* junction [30, 31]. If large quantities of lipids are removed during processing, particularly through the saponification of triglycerides during liming, this can produce voids facilitating the detachment—'delamination’—of the two layers.

Sheepskin has an inherently high lipid content, accounting for as much as 30–50% of the dermis dry weight, compared to 2–3% in cattle and 3–10% in goatskin [32–36]. Over half is saponified during liming [33, 37], with more removed during subsequent washing, shaving and degreasing steps. Consequently, the potential for scraping to delaminate these layers is considerably greater in sheepskin than those of other animals.

This increased visibility of textual manipulation is noted in the twelfth century *Dialogus de Scaccario*—attributed to Richard FitzNeal (1130–1189), Lord Treasurer during the reigns of Henry II and Richard I—which instructs scribes of the treasury to use of sheepskins “for they do not easily yield to erasure without the blemish being apparent” [38]. This sentiment prevailed into the seventeenth century, when Sir Edward Coke—Lord Chief Justice of the King’s Bench and foremost jurist of the early modern era—noted in his seminal *Institutes of the Laws of England* the necessity that deeds were written on a durable material such as parchment “for the writing upon these is least liable to alterations or corruption” [9]. Wakelin’s [39] survey of scribal correction found that in Tudor Royal accounts (documents likely written on sheepskin parchment) errors during writing were not scraped away and overwritten but crossed out and interlineation inserted between sentences, perhaps in acknowledgement of this risk.

The greater visibility of textual erasure afforded by sheepskin was undoubtedly a factor in the development of this preference, but their long-lasting predominance was likely due in no small part to their great abundance and relatively low cost. Estimating the size of the British sheep population prior to the introduction of official agricultural statistics has proved difficult [40, 41], however, it is likely that there were between 10 and 17 million sheep across the twelfth to seventeenth centuries, 11–14 million by the early eighteenth century and continuing to grow to over 25 million by the late-nineteenth century. With an average culling rate of around 20% during this period [42, 43], roughly 2–5 million skins would have been yielded annually, more than enough to meet the demands of British skin processors [42]. In contrast, the goat population of Britain has historically been very low [44, 45].

While sheepskins of any age can be used for parchment, only those from calves younger than around 6 weeks old can be used for vellum due to their rapidly increasing thickness [46]. The total number of calves is unlikely to have exceeded 1 million until the nineteenth century, of which only a few hundred thousand skins may have been yielded annually [47], particularly during the prohibiting of killing calves under 5 weeks between 1604 and 1671.^3^ The limited supply of calfskin, and its perceived higher quality, meant that vellum was more than double the price of sheepskin parchment [6–8]. Even the finest quality sheepskin was cheaper than the poorest quality vellum, as attested in the fourteenth century account books of Beaulieu Abbey [48]. In 1593, a dozen sheets of parchment cost on average 8*s*, while the same amount of vellum was more than double at 20*s*; by 1660, a dozen sheets of parchment cost 10*s* and vellum 28*s* [7], and more than double the levy of tax.^4^ Prior to the short-lived *Flaying Acts*^5^ (1800–1824), sheepskins were also exempt from the often costly inspection by ‘searchers and sealers’ which was required prior to processing for from calves, bulls, steers, deer and goats,^6^ likely making the former cheaper and more easily accessible. Consequently, for common legal documents, sheepskin parchment presented the ideal inexpensive and durable material.

## Conclusion

From the thirteenth to twentieth century, parchment legal deeds were almost exclusively written on sheepskin, rather than goatskin or calfskin vellum. This preferential use likely began due to the high fat content of sheepskins and their subsequent propensity to delaminate and deform when scraped, highlighting any attempts to modify the text after signing. Their abundance, low-cost and lower stamp duty throughout much of the early modern period supported the continuation of this practice through to at least late-nineteenth century.

While the text enshrined in these early modern deeds may be considered by some to be of limited historic value, as physical objects they are an extraordinarily high resolution zooarchaeological and molecular archive through which centuries of craft, trade and livestock economies can be explored. Once again, we have demonstrated how the growing field of ‘biocodicology’ [49] can bring life to the official archives and record offices, which Frederick Maitland [50] called the “mausoleum of parchment”.

## Supplementary Information

**Additional file 1: Dataset S1.** Scratching the Surface: the use of sheepskin parchment to deter textual erasure in early modern legal deeds

## Data Availability

Additional file 1: Dataset S1—Sample information and species identification.

## Footnotes

1 The requirement for formally executed instruments was particularly shaped by the Statutes of Uses, 1535 (27 Hen. 8, c.10), Statutes of Wills, 1540 (32 Hen. 8, c.1), and the Statutes of Frauds, 1677 (29 Car. 2, c.3) (1677).

2 The requirement that deeds be “written on paper or parchment” was formalised in the ruling of *Goddard’s Case* (1584) 2 Co Rep 4b, 5a; 76 ER 396, 398–9.

3 The prohibition was established by An Act Concerning Tanners, Curriers, Shoe Makers, and other artificers occupying the cutting of Leather, 1604 (1 Jac. I. c.22), but repealed by An Act to prevent Fraudes in the buying and selling of Cattell in Smithfeild and elsewhere, 1671, (22 & 23 Car. 2, c.19).

4 Taxation of skins for legal deeds was established by the *Stamps Act*, 1694 (9 Ann. c.11). The duties levied changed significantly over the proceeding centuries but all were repealed by the *Stamps Act*, 1815 (55 Geo. III c.184).

5 Skin inspections were created in 1804 (39 & 40 Geo 3, c. 6) and abolished in 1824 (5 Geo. 4, c. 57).

6 Although sheepskins were mentioned in the initial 1604 Act (1 Jac 1, c.22), the 1606 Act (4 Jac. 1, c.6) clarified that they were not subject to sealing.
